# Improvement of ECM-based bioroot regeneration via *N*-acetylcysteine-induced antioxidative effects

**DOI:** 10.1186/s13287-021-02237-5

**Published:** 2021-03-22

**Authors:** Jiayu Zhang, Tingting Lan, Xue Han, Yuchan Xu, Li Liao, Li Xie, Bo Yang, Weidong Tian, Weihua Guo

**Affiliations:** 1 State Key Laboratory of Oral Diseases, National Clinical Research Center for Oral Diseases, West China Hospital of Stomatology, Sichuan University, No. 14, 3rd Sec., Ren Min Nan Road, Chengdu, 610041 China; 2 Engineering Research Center of Oral Translational Medicine, Ministry of Education, West China Hospital of Stomatology, Sichuan University, Chengdu, China; 3 National Engineering Laboratory for Oral Regenerative Medicine, West China Hospital of Stomatology, Sichuan University, Chengdu, China; 4 Department of Pediatric Dentistry, West China Hospital of Stomatology, Sichuan University, No. 14, 3rd Sec., Ren Min Nan Road, Chengdu, 610041 China; 5 Department of Oral and Maxillofacial Surgery, West China Hospital of Stomatology, Sichuan University, Chengdu, China

**Keywords:** Dental follicle stem cell, Treated dentin matrix, Bioroot regeneration, *N*-Acetylcysteine, Oxidative stress

## Abstract

**Background:**

The low survival rate or dysfunction of extracellular matrix (ECM)-based engineered organs caused by the adverse effects of unfavourable local microenvironments on seed cell viability and stemness, especially the effects of excessive reactive oxygen species (ROS), prompted us to examine the importance of controlling oxidative damage for tissue transplantation and regeneration. We sought to improve the tolerance of seed cells to the transplant microenvironment via antioxidant pathways, thus promoting transplant efficiency and achieving better tissue regeneration.

**Methods:**

We improved the antioxidative properties of ECM-based bioroots with higher glutathione contents in dental follicle stem cells (DFCs) by pretreating cells or loading scaffolds with the antioxidant NAC. Additionally, we developed an in situ rat alveolar fossa implantation model to evaluate the long-term therapeutic effects of NAC in bioroot transplantation.

**Results:**

The results showed that NAC decreased H_2_O_2_-induced cellular damage and maintained the differentiation potential of DFCs. The transplantation experiments further verified that NAC protected the biological properties of DFCs by repressing replacement resorption or ankylosis, thus facilitating bioroot regeneration.

**Conclusions:**

The following findings suggest that NAC could significantly protect stem cell viability and stemness during oxidative stress and exert better and prolonged effects in bioroot intragrafts.

## Background

Tissue-engineered extracellular matrix (ECM)-based implants, despite some limitations, are gaining considerable attention in the field of regenerative medicine. The ECM provides structural strength to tissues, maintains a complex architecture around cells, and maintains the shapes of organs; thus, the ECM plays an important role in restoring damaged or lost tissues [1–3]. However, allogeneic or xenogeneic transplantation often leads to inflammatory and immune responses [4]. These leukocytes produce oxygen-derived free radicals that can directly damage graft cells via cell membrane damage. In addition, in the context of bacterial infection [5], ischaemia/reperfusion injury [6], hypoxia, or low nutrient supply [7], seed cells produce excessive intracellular reactive oxygen species (ROS), which lead to oxidative stress.

Appropriate stress responses are particularly important in stem cells, which provide lifetime support for tissue formation and repair, but excessive ROS can bind to and oxidize different cellular compounds (lipids, DNA, proteins, and cellular membranes, among others) [8], thus damaging cell structure and integrity and inhibiting cell adhesion [9]. In addition, ROS function as second messengers in the regulation of inflammatory mediator gene expression [10]. A high level of active oxygen also enhances the transformation of helper cells into inflammatory cells that is further increased with the decline of cell metabolism processes. Tissue necrosis caused by both oxidative stress and the inflammatory response further prevents nutrients and oxygen from reaching viable cells, which contributes to expanding the necrotic area and leads to implant failure [7]. Therefore, the unfavourable microenvironment after isolation and transplantation reduces the stemness and inhibits the therapeutic effects of surviving seed cells in intragrafts [11]. Thus, specific control of the balance within this cellular microenvironment may represent an efficient and novel strategy to increase the success rate of transplantation.

Teeth are complex organs that comprise ligament-to-bone attachments, which are complex hierarchical tissue structures that are amenable to tissue-engineering approaches that promote regeneration after injury [12, 13]. Our previous studies have proven that the combination of dental follicle stem cells (DFCs) and natural scaffold-treated dentin matrix (TDM) is a feasible strategy for bioengineered root regeneration [14–18]. DFCs, the precursor cells of periodontal tissues, represent a population of early stem/progenitor cells compared with other adult dental stem cells [19]. More importantly, DFCs can be easily obtained from impacted wisdom tooth and have self-modification capability for chromosome instability during freeze-thaw cycle or long-term culturing [20]. Thus, DFCs are considered suitable seed cells for bio-root construction. However, as in other transplantation processes, excessive ROS may cause poor viability of transplanted MSCs. These nonvital seed cells may lead to undesirable results, such as replacement resorption, ankylosis, or even osseous replacement [21], which hinders true tooth root regeneration that is different from implant osseointegration. This emphasizes the urgent need for delivering a multifunctional agent capable of improving the antioxidant capacity of seed cells, thus overcoming the low cellular survival and transdifferentiation potency to ultimately achieve biotooth regeneration.

*N*-Acetylcysteine (NAC) is a thiol compound with a small molecular weight, and it is a precursor of glutathione (GSH) [22, 23]. As an ROS scavenger, NAC regulates excessive inflammatory responses and prevents the oxidative stress triggered by ROS [24]. In addition, accumulating evidence has revealed that NAC exerts a wide range of pharmacological effects, such as induction of differentiation, antibacterial activity, and promotion of wound healing and osteogenic differentiation [5, 22, 25]. These findings indicate that NAC modifies biomaterials in many ways which may be distinct from that mediated by its well-known antioxidant effect [25], thus simultaneously overcoming various obstacles. Although various pretreatment strategies have been reported to mitigate the damage caused by adverse microenvironments, different pretreatments are needed for different organs or cell delivery methods.

It remains unclear whether NAC can exert a protective effect to facilitate bioroot regeneration. The length of survival time and the number of seed cells that remain active in allograft tissues still need to be determined. In this study, we improved the antioxidative properties of ECM-based bioroots by pretreating cells or loading scaffolds with the antioxidant NAC. Our results suggested that NAC could promote seed cell antioxidant capacity to protect against redox imbalances and improve the engraftment efficiency of bioengineered roots.

## Methods

### TDM preparation

This research was approved by the Ethics Committee of West China College of Stomatology, Sichuan University, China (WCHSIRB-D-2019-063, WCHSIRB-D-2019-065). Human samples were collected from freshly extracted teeth with the informed consents of the donors or their guardians.

Treated dentin matrices (TDMs) were prepared using a previously described method [26]. Briefly, rTDMs were obtained from the mesial tooth root of the first molars of adult SD rats, and hTDMs were obtained from human premolars freshly extracted for orthodontic or periodontal reasons. Then, the periodontal ligament, outer cementum, and partial root dentins were gently removed using a dental handpiece. Next, the dentin matrix was ultrasonicated, washed with deionized water, and demineralized with gradient ethylenediaminetetraacetic acid (EDTA, Sigma-Aldrich, St. Louis, MO, USA). The obtained TDMs were freeze-dried by a Vacuum Freeze Drier (Biocool, Beijing, China) and sterilized with ethylene oxide. The rTDMs were prepared for transplantation. The hTDMs were ground into small particles with a ball mill, and the extractions were used for subsequent in vitro experiments.

### In vitro protein release study

The BCA assay method (KeyGen Biotech, Nanjing, China) was performed to detect the total protein concentration released by 1 g of hTDM particles soaked in 5 mL saline. The absorbance of the solution at different time points (4 h, 8 h, 1 day, 3 days, and 7 days) was measured. At each time point, the extracts were collected from each well, the total protein concentration was detected, and then, the extracts were replaced in 5 mL fresh saline.

### Cell culture and identification

DFCs were isolated and cultured, as previously described [26]. hDFCs were obtained from freshly extracted third molars of healthy, young individuals under 18 years of age. rDFCs were harvested from Sprague-Dawley rat pups aged 3–5 days and cultured. The cellular ultrastructure was assessed with electron microscopy. Furthermore, the cellular multipotent osteogenic, adipogenic, and neural differentiation potential was determined as described below.

hDFCs of passages 3–5 were used for cytology experiments. The cells (1 × 10^4^) were seeded in 12-well cell culture plates for multipotential differentiation. After reaching 80% confluence, the hDFCs were cultured in osteogenic/adipogenic/neuronal induction medium (osteogenic induction medium: 10% FBS (HyClone, Waltham, MA, USA), 10 nM dexamethasone (Sigma-Aldrich), 50 μg/ml Vitamin C (Sigma-Aldrich), 0.01 μM 1,25-dihydroxyvitamin D3 (Sigma-Aldrich), and 10 mM β-glycerol phosphate (Sigma-Aldrich); adipogenic induction medium: 10% FBS (HyClone), 111 μg/ml isobutyl-methylxanthine (IBMX; Sigma-Aldrich), 72 μg/ml indomethacin (Sigma-Aldrich), 5 μg/ml insulin (Novo Nordisk, Tianjing, China), and 0.4 μg/ml dexamethasone (Sigma-Aldrich); neuronal induction medium: 2% DMSO (Amresco), 200 μM butylated hydroxyanisole (Sigma-Aldrich), 25 mM KCl (bdhg Company, Tianjing, China), 2 mM valporic acid sodium salt (Sigma-Aldrich), 10 mM forskolin (Sigma-Aldrich), 1 mM hydrocortisone (Aladdin, Shanghai, China), and 5 μg/ml insulin (Novo Nordisk)). The medium was changed every 2–3 days. After 14 days of culture in osteogenic induction medium, 14 days of culture in adipogenic induction medium, or 2 h of culture in neuronal induction medium, the cells were fixed in 4% paraformaldehyde and stained with Alizarin Red S, Oil Red O, and βIII-Tubulin antibodies, respectively.

### Cell treatment and cell proliferation assay

The cell viability was assessed by the CCK-8 method. Cells of passages 3–5 were used in the experiments. hDFCs were seeded in 96-well plates at a density of 8 × 10^3^ cells per well. H_2_O_2_ (Sigma-Aldrich), NAC (Beyotime Biotechnology, Shanghai, China), or hTDM extract was separately added to the culture medium in different concentration gradients. After cultivation for 24 h, the culture medium was replaced with 100 μL α-MEM medium and 10% (v/v) CCK-8 solution per well. After 1.5 h of incubation at 37 °C, the absorbance was measured at 450 nm using an absorbance microplate reader. To assess the effect of NAC in reversing the reduction of cell viability induced by H_2_O_2_, the hDFCs were pretreated with different concentrations of NAC for 24 h before addition of H_2_O_2_ for 24 h. After treatment, the cell viability was examined by the CCK-8 method. To assess the effect of NAC on the proliferation of DFCs for 1 week, hDFCs were seeded in 96-well plates at a density of 2 × 10^3^ cells per well and treated with different concentrations of NAC. CCK8 assays were performed once a day.

### Intracellular ROS and glutathione production

The intracellular ROS levels were measured by 2′,7′-dichlorofluorescin diacetate (Sigma-Aldrich). hDFCs were pretreated with hTDM extract or NAC for 24 h as described above before addition of 100 μM H_2_O_2_ for 3 h, and then incubated with 20 μM DCFH-DA for 30 min at 37 °C. The cells were washed with PBS, and the fluorescence was immediately examined with a fluorescence microscope (Olympus Corporation, Tokyo, Japan). The content of intracellular reduced glutathione (GSH) was assessed using a reduced glutathione (GSH) assay kit (Jiancheng Bioengineering Institute, Nanjing, China). The intensity of the colorimetric reaction was determined by spectrophotometry at 405 nm.

### The effect of ROS on the migration of hDFCs

Cell migration was assessed using a Chemotaxicell chamber (8-μm pore size, Corning, China). The cells were resuspended and seeded in the upper chamber at a density of 8000 cells per well and then treated as described above. Following the different treatments, the medium in the upper chamber was replaced with α-MEM supplemented with 5% FBS, and the medium in the lower chamber was replaced with α-MEM supplemented with 20% FBS. Then, the plates were incubated for 24 h. The cells that migrated to the lower surface of the membrane were fixed with 4% paraformaldehyde and stained with crystal violet staining solution. The experiment was repeated three times.

### The effect of ROS on the osteogenic differentiation of hDFCs

To evaluate the effect of ROS on the osteogenic differentiation of hDFCs, a total of 1 × 10^4^ hDFCs were seeded into 12-well plates in triplicate. The hDFCs were treated as described above. Following the different treatments, the cells were cultured in osteogenic induction medium containing 10% FBS, 10 nM dexamethasone, 50 μg/ml Vitamin C, 0.01 μM 1,25-dihydroxyvitamin D3, and 10 mM β-glycerol phosphate. The medium with its respective treatment solution was replaced every 3 days. Cells not treated with hTDM extract were used as the control group. Gene expression was analysed using reverse transcription-polymerase chain reaction (RT-PCR) on culture day 7. The mRNA expression of osteogenesis differentiation markers, such as ALP, COL1, RunX2, and Periostin, was detected with the relevant primers (Table 1). The expression levels of all the target genes were normalized to the expression level of GAPDH. Fourteen days later, the cells were stained with 2% Alizarin Red solution (Sigma-Aldrich) and photographed under a phase-contrast inverted microscope (Nikon, Tokyo, Japan).

**Table 1:** Primer sequences used for RT-PCR gene expression analysis

Target cDNA	Primer sequence (5′-3′)
Gapdh	CTTTGGTATCGTGGAAGGACTC GTAGAGGCAGGGATGATGTTCT
ALP	TAAGGACATCGCCTACCAGCTC TCTTCCAGGTGTCAACGAGGT
COL1	AACATGGAGACTGGTGAGACCT CGCCATACTCGAACTGGAATC
RunX2	CTTTACTTACACCCCGCCAGTC AGAGATATGGAGTGCTGCTGGTC
Periostin	CACTCTTTGCTCCCACCAATA ATTTCCTTCCAGCGTCTCAA

### Construction of cell sheets

For the preparation of DFC sheets (DFCSs), rDFCs were seeded in 24-well plates. When the cells reached 70% confluence, 50 μg/mL ascorbic acid (Sigma-Aldrich) was added to the culture medium to enhance extracellular matrix formation [12]. The medium was changed every 48 h. After an additional 2 weeks of culture, the rDFCSs were harvested and prepared for subsequent animal surgery.

### Animal surgery

#### Transplantation of bioroot composites in a bone defect model

A total of 8 female Sprague-Dawley (SD) rats (8 weeks old) were obtained from the Animal Experiment Center of Sichuan University and housed under temperature- and humidity-controlled conditions with a 12-h light/dark cycle. The left and right sides of the mandible were divided into 2 groups (*n* = 8 per group): (1) rTDM/rDFCSs and (2) rTDM/ rDFCSs/NAC. Twenty-four hours before transplantation, the rDFCSs and rTDM scaffolds in the NAC-treated group were pretreated with 5 mmol NAC. The permeability of the TDM scaffold allows the sustained release of growth factors through its porous structure to form a concentration gradient at the TDM interface [15]. To trace the rDFCs in vivo, all the transplanted cells were labelled with green florescent protein (GFP) through viral transduction at an MOI of 40.

For animal surgery, transplants were implanted into the defects drilled into the surface of the mandible with a critical size of 2 mm × 2 mm after the rats were anaesthetized with 10% chloral hydrate (2 ml kg^−1^) and Zoletil 50 (50 mg kg^−1^). Then, the muscles and skins were carefully sutured, and the animals received antibiotics immediately after surgery. Then, to examine the survival of the cells in the implants, 4 rats were sacrificed on day 1 and day 3. The implants were harvested and stained with propidium iodide (PI, KeyGen Biotech) to visualize the dead cells; PI is able to pass through the damaged membranes of dead cells to produce red fluorescence in the nucleus.

#### Transplantation of bioroot composites in rat alveolar fossa

Eight-week-old female Sprague-Dawley rats were randomly divided into 3 groups: (1) rTDM, (2) rTDM/rDFCSs, and (3) rTDM/rDFCSs/NAC. The adult rats were anaesthetized, and their oral cavities were disinfected. Next, the bilateral maxillary first molars were removed, exposing the alveolar fossa. Then, transplants pretreated as described above were implanted into the tooth extraction sockets in the upper first molar region of the alveolar fossa. Then, the incised oral mucosa was sutured with 6–0 nylon. In addition, the surgical site was cleaned.

Two rats from each group were sacrificed at 1 week or 2 months postoperatively. The maxilla were harvested and fixed with 4% paraformaldehyde at 4 °C overnight, decalcified using 10% EDTA (pH 8.0), and embedded in paraffin. For histological analysis, paraffin sections (5 mm) were stained with haematoxylin and eosin (H&E), Masson’s trichrome, and TRAP/ALP staining kits, according to the manufacturer’s recommended protocol.

## Results

### Characterization of DFCs, establishment of H_2_O_2_-induced oxidative stress model, and determination of certain NAC treatment doses

**Fig. 1:**
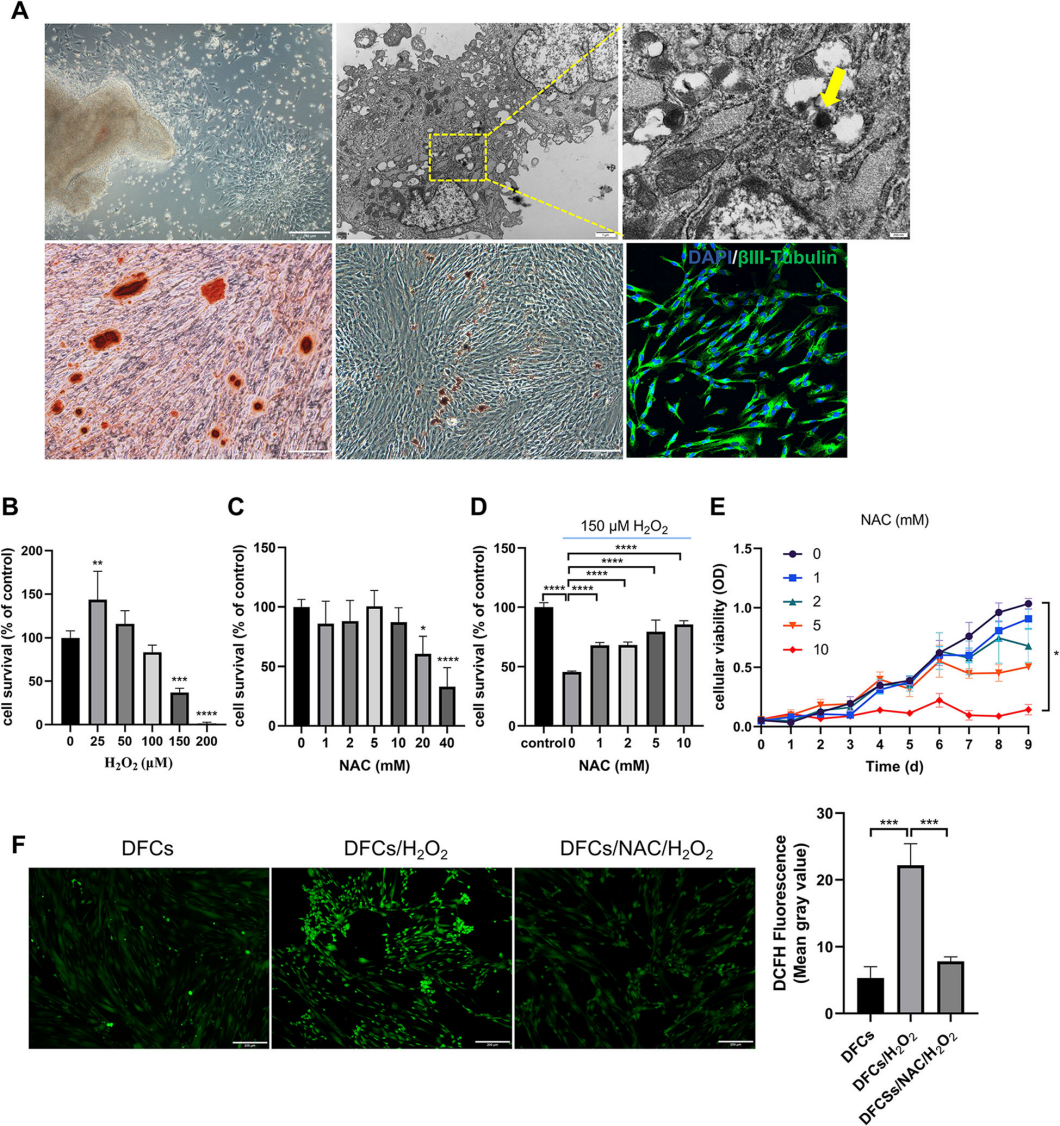
Characterization of DFCs, establishment of H_2_O_2_-induced oxidative stress model, and determination of particular NAC treatment dose. **a** Characterization of DFCs. Primary DFCs were adherent to plastic and showed colony-forming abilities. TEM evaluations showed homogeneous electron-dense granules without membranous structures (indicated by yellow arrow). After being cultured in osteogenesis and adipogenesis-inducing media for 14 days and in neuronal-inducing medium for 2 h, the cultured DFCs showed mineralized nodules, lipid clusters, and positive βIII-tubulin expression, respectively. **b** Effect of different concentrations of H_2_O_2_ on the viability of hDFCs. **c** Effect of different concentrations of NAC on the viability of hDFCs. **d** Effect of NAC on the viability of hDFCs under oxidative stress conditions induced by 150 μm H_2_O_2_. **e** Effect of NAC on the proliferation of hDFCs for 1 week. **f** Assessment of ROS levels using fluorescence microscopy and statistical analysis of DCFH fluorescence from three experiments. Asterisks indicate statistically significant differences compared with the control group. ∗*P* < 0.05, ∗∗*P* < 0*.*01, and ∗∗∗*P* < 0*.*001

The isolated cells were adherent to plastic and exhibited colony-forming abilities. TEM evaluations showed homogeneous electron-dense granules without membranous structures (Fig. 1a, indicated by yellow arrow), which are considered identifying markers of DFCs [26]. After being cultured in adipogenesis/osteogenesis-inducing medium for 14 days, the DFCs differentiated into adipocytes and osteoblasts, respectively, which was confirmed by positive staining with Oil Red O and Alizarin Red, respectively. After being cultured in neuronal induction medium for 2 h, the DFCs showed positive βIII-tubulin expression (Fig. 1a). These results confirmed the stem cell characteristics of dental follicle cells.

To determine the appropriate drug concentrations, we performed a CCK-8 assay to measure the viability of hDFCs after they were incubated with different concentrations of H_2_O_2_ and NAC. As shown in Fig. 1b, the survival of the hDFCs showed a trend of first increasing and then decreasing, and the survival significantly decreased following treatment with concentrations of H_2_O_2_ above 100 μM; this result was consistent with previous studies showing that high ROS levels cause cellular damage and dysfunction, but low basal ROS levels are necessary and advantageous for maintaining cellular proliferation, differentiation, and survival [27]. Considering that cell death could interfere with the ROS and GSH measurements [28], 100 μmol/L H_2_O_2_ was used as the sublethal dose for the following experiments designed to establish the oxidative stress model. Then, the viability of the hDFCs treated with different concentrations of NAC was measured, and cytotoxicity was observed at drug concentrations greater than 10 mM (Fig. 1c). To investigate whether NAC ameliorates the H_2_O_2_-induced cytotoxicity, hDFCs were incubated with 150 μM H_2_O_2_ after pretreatment with different concentrations of NAC. The results showed that NAC exerted a protective effect against H_2_O_2_-induced cytotoxicity in hDFCs; the effect is dose-dependent and peaked at a concentration of 10 mM (Fig. 1d). However, the effect of NAC on the proliferation of DFCs for 1 week revealed that NAC could inhibit the proliferation of hDFCs in an almost dose-dependent manner. Compared with the 0 mM NAC group, the 10 mM NAC group has significant difference, with statistical significance (Fig. 1e). And cells growth in lower NAC concentrations slowly proceeded with incubation time. In addition, fluorescence microscopy images showed that pretreatment with 5 mM NAC significantly reduced the high level of intracellular ROS (*p* < 0.05) down to the level observed in the control hDFC group (Fig. 1e). Based on these results, 5 mM NAC was chosen for the subsequent experiments.

### NAC attenuated the intracellular oxidative stress status in vitro

**Fig. 2:**
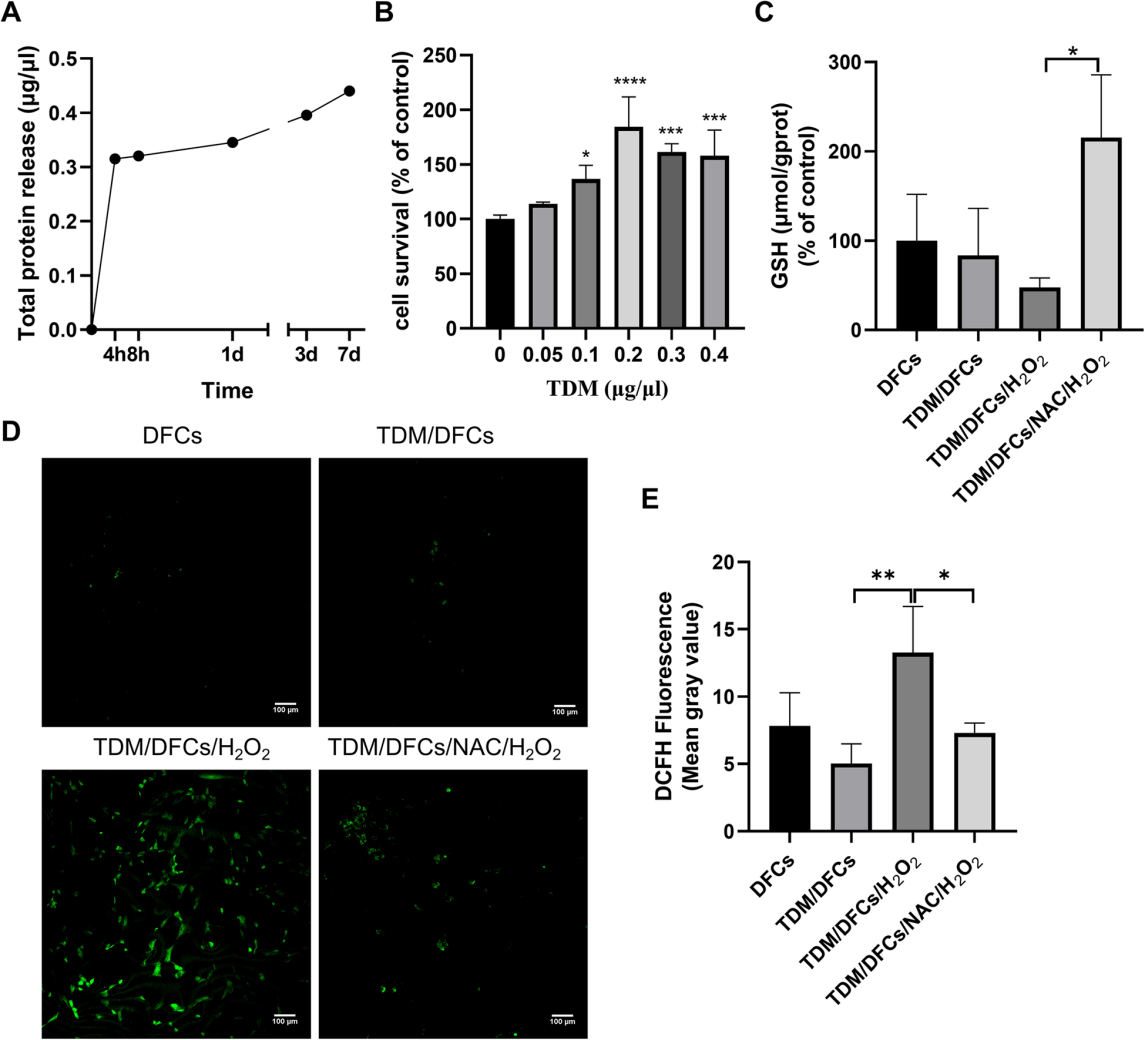
The effect of NAC and the H_2_O_2_-induced intracellular oxidative stress status on TDM/DFCs system. **a** Protein release of the TDM scaffold. **b** Effect of different concentrations of hTDM protein on the viability of hDFCs. **c** GSH level. **d** The level of ROS in hDFCs was measured by DCFH-DA with a fluorescence microscope. **e** statistical analysis of (**d**)

In previous studies, we identified treated dentin matrix (TDM) as a natural scaffold material for regenerating bioengineered roots. To evaluate the effect of scaffolds on the survival of DFCs and to mimic the in vivo transplantation microenvironment, the scaffold protein release and cell viability after being cocultured with different concentrations of protein were evaluated by CCK-8. The protein release profiles of the TDM particles are shown in Fig. 2a. For the first 4 h, most of the total protein was released from the TDM. There was sustained release of the protein from day 1 to day 7. The total protein release was related to the particle size of the TDM (data not shown). The CCK-8 assay results showed that the TDM scaffold was favourable for cell viability. The protective and growth-promoting effects of TDM peaked at a concentration of 0.2 μg/μL (Fig. 2b). Based on these results, 0.2 μg/μL TDM extract was chosen for cell coculture experiments to eliminate H_2_O_2_-induced cytotoxicity and to further investigate the role of the oxidative stress environment in the TDM/DFCs system.

To ensure the effects of NAC and the H_2_O_2_-induced intracellular oxidative stress status on the TDM/DFCs system, we assessed the levels of intracellular ROS and reduced glutathione in the hDFCs. The intracellular ROS production in the hDFCs experiencing oxidative stress was greater than that in the cells cultured with TDM extract without H_2_O_2_ (Fig. 2d, e) (*p* < 0.05). Pretreatment with 5 mmol/L NAC significantly reduced the level of intracellular ROS (*p* < 0.05) to the level observed in the control hDFCs (*p* > 0.05). The cells cultured with 100 μmol/L H_2_O_2_ showed a decreased cellular concentration of the antioxidant glutathione (GSH), indicating the consumption of GSH by ROS. Pretreatment with NAC completely restored the H_2_O_2_-induced depletion of reduced GSH (*p* < 0.05) and increased the glutathione levels to levels higher than those observed in the control hDFCs (Fig. 2c).

### NAC alleviated the suppression of H_2_O_2_-induced cell differentiation

**Fig. 3:**
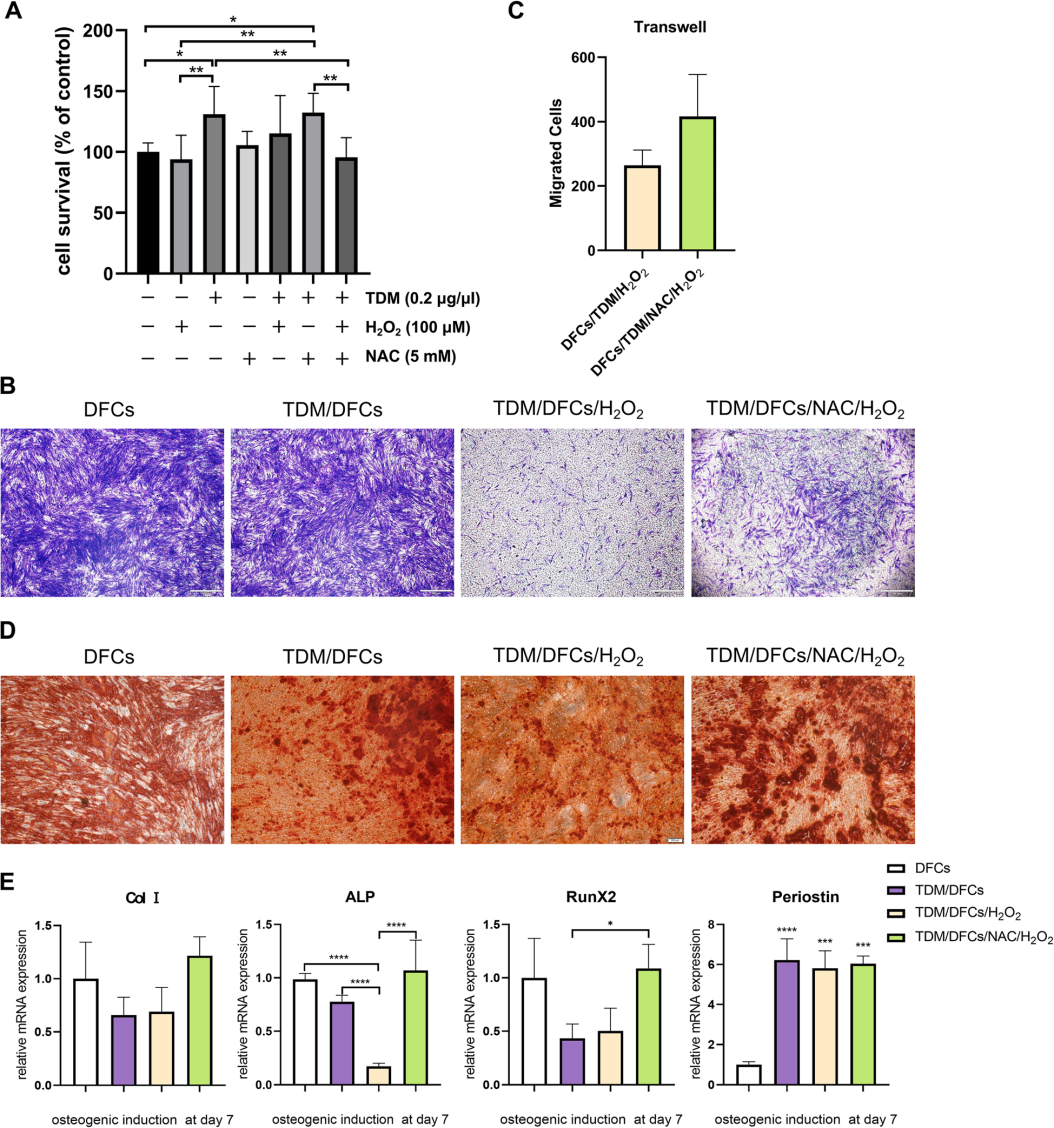
The effect of NAC on the proliferation, migration and osteogenic differentiation of hDFCs under H_2_O_2_-induced oxidative stress. **a** Effect of combination drug treatment on the viability of hDFCs. **b** After 24 h of culture in Transwell plates, crystal violet staining was performed. **c** Pretreatment with NAC led to increased hDFC migration to the opposite side of the membrane compared with that observed in the H_2_O_2_-treated groups. **d** The effect of NAC on the osteogenic differentiation of hDFCs stimulated with H_2_O_2_, as determined by Alizarin Red staining. **e** RT-PCR analysis of Collagen I, ALP, RunX2, and Periostin mRNA expression. ∗*P* < 0.05, ∗∗*P* < 0*.*01, and ∗∗∗*P* < 0*.*001

We further conducted in vitro experiments to verify the role of NAC in the proliferation, migration, and osteogenic differentiation of hDFCs under H_2_O_2_-induced oxidative stress. Because 100 μmol/L H_2_O_2_ was used as the sublethal dose to establish the oxidative stress model, the CCK-8 assay showed no obvious cytotoxicity or proliferation-promoting effect on the DFCs when TDM, NAC, and H_2_O_2_ were administered in combination (Fig. 3a). Then, Transwell assays showed a significant reduction in cell migration in the H_2_O_2_-treated groups (TDM/DFCs/H_2_O_2_ and TDM/DFCs/NAC/H_2_O_2_) compared to that in the untreated groups (DFCs and TDM/DFCs). However, pretreatment with NAC for 24 h resulted in a nonsignificant reduction in cell migration, but NAC pretreatment was unable to restore the cell migration to the levels observed in the cells not treated with H_2_O_2_ (Fig. 3b, c).

In mineral nodule formation (Fig. 3d) and mRNA expression (Fig. 3e), similar and clear trends were observed. On day 14, the differentiation-induced hDFCs cultured in osteogenic medium showed sparser and fewer mineralized nodules in response to H_2_O_2_ stimulation. However, in the same H_2_O_2_-induced oxidative stress model, more dense and intense mineralized nodules were observed in the NAC-treated culture than in the untreated. The expression of genes in the bone matrix, including the expression of collagen I, ALP, and RunX2 in the osteoblastic culture, was increased in the groups treated with both NAC and H_2_O_2_ compared to the groups treated with H_2_O_2_ alone. Periostin was markedly upregulated in the TDM group compared with the control group. TDM extract dramatically reduced the inhibitory effect of oxidative stress on the odontogenic differentiation of the DFCs. These results demonstrated that in the context of H_2_O_2_-induced oxidative stress, NAC protected the osteogenic potential of hDFCs, which is one of the most important characteristics of the stemnness of DFCs.

### NAC pretreatment increased the survival rates of the transplanted cells in vivo

**Fig. 4:**
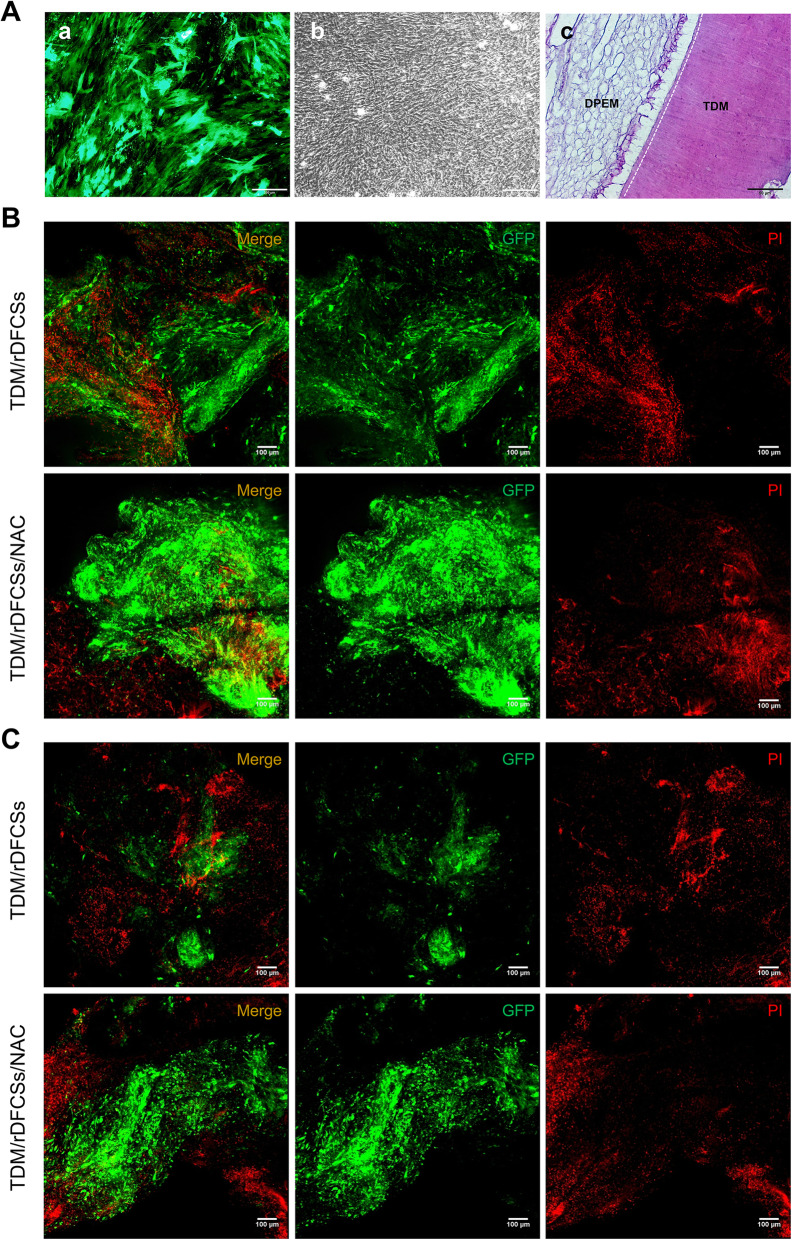
Allogeneic transplantation of bioroot composites in a Sprague-Dawley rat bone defect model for 8 weeks. **A** (a) GFP-labelled rDFCs. **A** (b) Construction of cell sheets. **A** (c) Histological section of the TDM scaffold combined with intrinsic fibre three-dimensional dental pulp extracellular matrix, as determined by haematoxylin and eosin (HE) staining. Images of PI staining to detect the survival of grafted cells on **B** day 1 postimplantation and **C** day 3 postimplantation. TDM, treated dentin matrix; DPEM, dental pulp extracellular matrix

Bioroot composites are implanted into injured sites, and seed cells are thus placed in a relatively harsh environment. Therefore, the length of time and the number of DFCs that remain active in allograft tissue are issues that should be taken into consideration. All the cells used in this experiment were labelled with green florescent protein (GFP), which was used to distinguish between the engrafted cells and endogenous cells. The successfully transfected rDFCs expressed GFP and exhibited green fluorescence (Fig. 4A (a)). The addition of ascorbic acid to the cell culture media resulted in the deposition of a well-developed collagenous network in a closely packed cell structure (Fig. 4A (b)) [29]. The edge of the mature sheets began to curl after 2 weeks of culture, and they were thick enough to wrap around the rTDM scaffold.

After implanting the allograft rTDM scaffolds wrapped with GFP-labelled rDFC membrane sheets into the intramedullary cavity of the rat mandible for 1 day and 3 days, we evaluated the survival rates of the transplanted cells by staining with propidium iodide (PI). Confocal microscopy was used to obtain images showing the 3-dimensional morphology of the DFC sheets under 2 conditions: (a) pretreatment with NAC for 24 h (rTDM/rDFCSs/NAC) and (b) without pretreatment (rTDM/rDFCSs). The results indicated that both groups showed similar growth trends in the acute inflammatory stage. On day 1 (Fig. 4B), the GFP-labelled cells exhibited spindle-shaped morphologies and showed tight arrangements with multilayers. The cells with overlapping red and green fluorescence signals represented dead implanted cells. Compared with those in the untreated group, the survival rates of the implanted cells in the NAC pretreated group were significantly higher. Although the number of cells emitting green fluorescence on the scaffold surface gradually decreased in the acute inflammatory stage, an increase in the number of remaining cells was observed in the NAC-treated group compared to the untreated group throughout this process (Fig. 4B, C).

### NAC pretreatment improved the engraftment efficiency of TDM/DFCSs composites in vivo

**Fig. 5:**
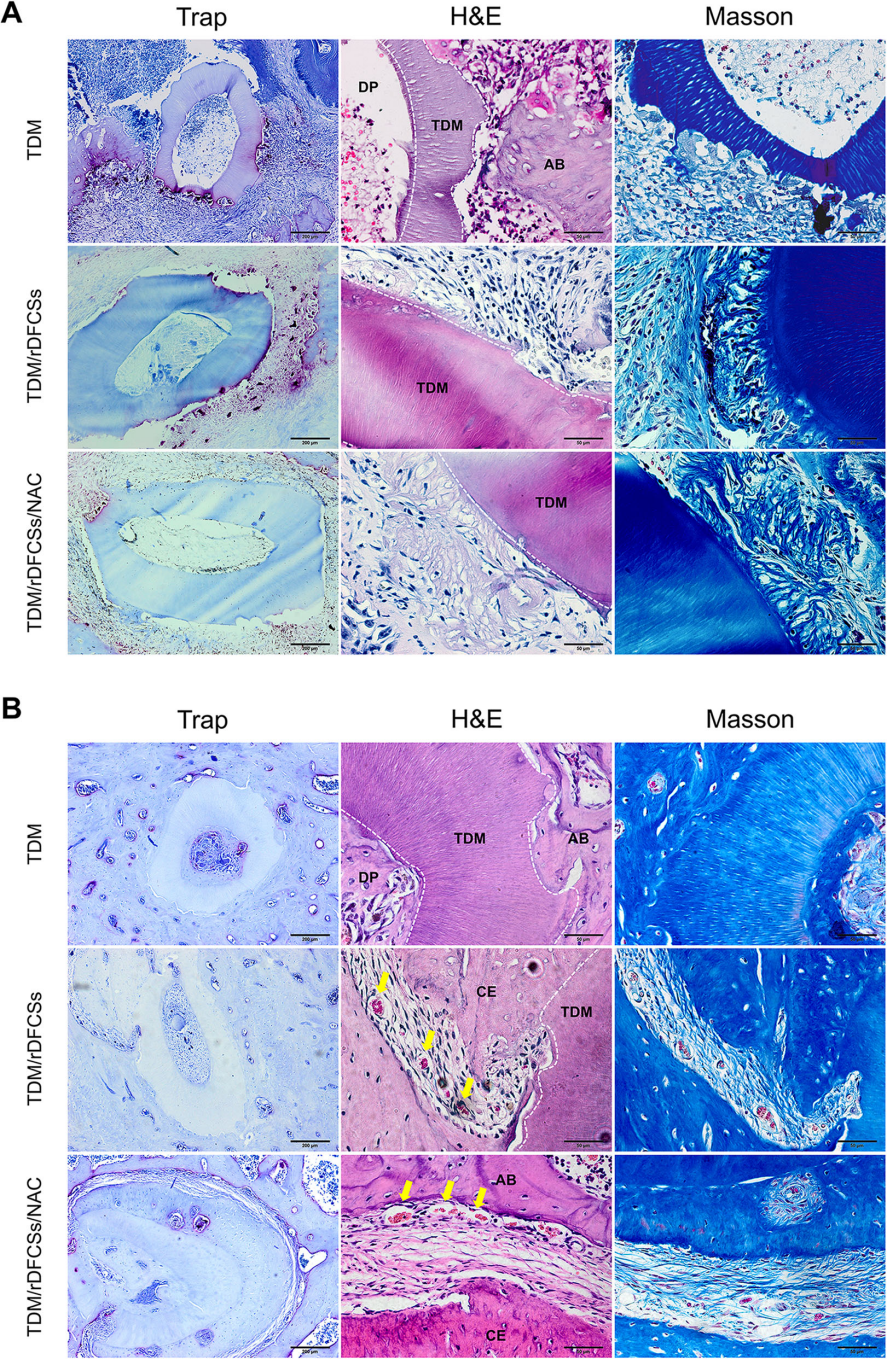
Allogeneic transplantation of bioroot composites in the alveolar fossa of Sprague-Dawley rats for 8 weeks. Images of TRAP, HE, and Masson staining to detect the efficiency of transplantation at **a** 1 week postimplantation and **b** 2 months postimplantation. TDM, treated dentin matrix; AB, alveolar bone; CE, cementum; DP, dental pulp; margin of the TDM scaffold (white dashed line); neovascularization (yellow arrows)

In the acute inflammatory phase (1 week), inflammatory cells and osteoclasts were dispersed on the periphery of the TDM scaffold and the adjacent alveolar bone. Obvious resorption of the scaffold was detected at the site where the osteoclasts accumulated, especially in the two groups without NAC pretreatment (Fig. 5a). Additionally, an unorganized fibre structure with no clear direction was observed at the periphery of the scaffold in the groups implanted with cell sheets (rTDM/rDFCSs, and rTDM/rDFCSs/NAC groups). No internal root resorption lacunae were observed in any TDM scaffold. Large accumulations of extravasated erythrocytes and occasional inflammatory cells were evident.

After 2 months (Fig. 5b), the rTDM alone group showed decreased osteoclastogenesis/osteoclast activity. Osseous replacement was reached very deep into the dentin, and large areas of dentin were lost. Significantly more dentin remained in the rTDM/rDFCSs group, than in the rTDM alone group. The histological images of the peripheral scaffold showed local areas of fibre structures and ankylosis with osseous replacement. This may be because the adverse microenvironment reduces the stemness and inhibits the therapeutic effects of seed cells, similar to the case of delayed replantation of an avulsed tooth whose PDL cells are necrotic after long periods of dry extra oral time. In such teeth, ankylosis and osseous replacement are unavoidable [30]. In the rTDM/ rDFCSs/NAC group, significant cellular cementum-like tissues and fibre structures, including dense collagen fibres, fibroblasts, and blood vessels, were observed at the periphery of the TDM scaffold.

## Discussion

Mesenchymal stem cells (MSCs) are emerging as major seed cells for tissue engineering and cell-based therapies for several lung diseases, myocardial repair, large bone defects, and chronic wounds [9, 28, 31, 32] due to their pleiotropic properties, including their self-renewal ability, multidifferentiation potential, paracrine actions, and immunoregulatory ability [33]. To overcome the low cellular survival and transdifferentiation potential after implantation, various strategies for the pretreatment of intragrafts have been reported, and these strategies mainly include pretreatment with growth factors or cytokines, preconditioning such as hypoxia, and genetic modifications [9]. Enlightened by the spatial interface gradient from biomaterial scaffolds (TDMs) [15], this study reports a strategy for pretreating scaffolds and seed cells with antioxidant drugs to modulate the transplant microenvironment and promote the seed cell antioxidant capacity. In this study, we found that rTDM/ rDFCSs/NAC composites displayed a decrease in replacement resorption and an increase in cellular survival and transdifferentiation potency.

NAC reduces the cellular oxidative stress response by scavenging free radicals, thus restoring cell viability and protecting cells from apoptosis [34]. Ex vivo NAC preconditioning has been proven to be an effective strategy for improving post-transplantation survival rates [28, 35]. We found that 5 mmol/L NAC alleviated the H_2_O_2_-induced intracellular oxidative stress in hDFCs by reducing the production of ROS and promoting the synthesis of reduced GSH, an endogenous antioxidant that plays the most important role in maintaining cellular redox homeostasis. The antioxidant capacity of NAC-pretreated hDFCs was also assessed using cell differentiation and spreading assays. With regard to osteogenic differentiation, most studies suggest that ROS inhibit osteogenic differentiation [27]. In this study, we found that NAC increased the osteogenic differentiation of hDFCs under oxidative stress, promoting mineralized nodule deposition and Col I, ALP, RunX2 expression, which is consistent with a previous study [11]. The mRNA expression pattern of osteoblast-related genes is characteristic of the process of osteogenic differentiation. Collagen I and ALP are early markers of osteogenic differentiation [36]. RunX2/cbfa1 is an important transcription factor that plays a crucial role in the formation of the mineralized skeleton during embryogenesis and regulates the differentiation of osteogenic lineages [22]. Moreover, NAC itself can function as an osteogenesis-enhancing molecule to accelerate bone regeneration by activating the differentiation of osteogenic lineages [22]. Thus, there may be many reasons why NAC increased cell osteogenic differentiation under oxidative stress. Furthermore, the addition of TDM extracts to the osteogenic inducing medium in the present study significantly increased the gene expression of Periostin, an important regulator of periodontal tissue formation [37], and decreased the expression of Col I, ALP, RunX2. A possible reason is that that the basic component of TDM was far more complex than that of synthetic hydroxyapatite (HA); thus, it can influence the differentiation state of DFCSs [38]. When implanted into a non-mineralized mouse subcutaneous pocket, DFCSs/TDM were found to have little effect on osteogenesis, but contributed to the formation of dentin (predentin and odontoblast layer), and the pulp (fibroblasts and blood vessels) [14]. These results further illustrated that TDM may provide an inductive microenvironment for the construction of the tooth root rather than promotion of bone formation.

The utilization of an acellular matrix is currently a hot research topic in the field of tissue engineering, with the goal of harnessing the bioactivity of the retained extracellular matrix without the potentially negative immunogenic effects of the cellular material [2, 3]. TDM, which retains the important dentin matrix structure (dentinal tubules) and bioactive molecules, factors, proteins related to dentin and pulp tissue formation, has been shown to be an ideal dental scaffold material [39]. However, for the regeneration of organs with multiple and complex histological structures, simply utilizing a single scaffold material could hardly mimic all these complex ECM microenvironments [39]. In this study, we retained the network structure of dental pulp tissues and removed the cellular components through EDTA treatments and ultrasonication. Then, the TDM combined with intrinsic fibre three-dimensional dental pulp extracellular matrix (DPEM) was used as a biological scaffold for root regeneration in vivo.

The findings from a study of a bone defect model revealed that NAC-pretreated bioroots increased the survival rates of engrafted cells in the acute phase. A similar result was observed in a previous experiment [28]. In alveolar fossa implantation, the rTDM/ rDFCSs/NAC group showed less osteoclast formation in the acute phase and much more bone and fibre structure regeneration at 2 months posttransplantation. Regarding the activation of osteoclasts around the implants, previous studies noted that bone-resorbing cells require prior activation or continuous stimulation by factors such as necrotic debris and inflammation before resorption can proceed [21]. Notably, NAC has been reported to alleviate cellular inflammatory responses and exhibit anti-inflammatory activity by regulating the synthesis of proinflammatory cytokines [5, 34]. Thus, it is suggested that NAC pretreatment not only protected seed cells against apoptosis but also protected scaffolds from absorption, thus maintaining the integrity of the scaffold and achieving better bioroot regeneration. But our study still had limitations and can be further improved in the future. Given that most cell death occurs in the first hours to days after transplantation [40], the drug release observed in TDM that lasted several days can exert a protective effect on biological properties of DFCs in the acute phase. However, the slow and controlled release systems can increase the duration of local agents to different degrees, starting from prolongation by several hours to weeks, thus having been extensively explored for improving the delivery efficiency [41]. Whether slow-release or controlled-release delivery system combined with TDM scaffold is beneficial for further protecting transplanted stem cells requires further evaluation.

Multiple findings within this study indicate that this novel design may serve as a therapeutic treatment by improving the antioxidative properties of ECM-based implants to protect the biological properties of seed cells. And this design may be instructive for biomaterial construction strategies for bone defect restoration and repair following infection or inflammation.

## Conclusions

In conclusion, we have shown that NAC could protect the biological properties of seed cells by eliminating cellular ROS, increasing cellular glutathione levels, enhancing cellular osteogenic differentiation capacities, and improving cell antioxidant capacities to defend against redox imbalances after exposure to unfavourable transplant microenvironments Thus, NAC could increase transplant efficiency and achieve better ECM-based bioroot regeneration.

## Data Availability

The datasets used and/or analysed during the current study are available from the corresponding author on reasonable request.

## Abbreviations

ECM: Extracellular matrix
ROS: Reactive oxygen species
MSCs: Mesenchymal stem cells
DFCs: Dental follicle stem cells
DFCSs: Dental follicle stem cell sheets
TDM: Treated dentin matrix
NAC: *N*-Acetylcysteine
GSH: Glutathione
SD: Sprague-Dawley
GFP: Green florescent protein
PI: Propidium iodide
H&E: Haematoxylin and eosin
DPEM: Dental pulp extracellular matrix
